# High canopy cover of invasive *Acer negundo* L. affects ground vegetation taxonomic richness

**DOI:** 10.1038/s41598-021-00258-x

**Published:** 2021-10-21

**Authors:** D. V. Veselkin, D. I. Dubrovin, L. A. Pustovalova

**Affiliations:** Institute of Plant and Animal Ecology, Ural Branch, Russian Academy of Sciences, Yekaterinburg, Russia

**Keywords:** Ecology, Biodiversity, Community ecology, Invasive species, Urban ecology

## Abstract

We assessed the link between canopy cover degree and ground vegetation taxonomic richness under alien ash-leaved maple (*Acer negundo*) and other (native or alien) tree species. We investigated urban and suburban forests in the large city of Yekaterinburg, Russia. Forests were evaluated on two spatial scales. Through an inter-habitat comparison we recorded canopy cover and plant taxonomic richness among 13 sample plots of 20 × 20 m where *A*. *negundo* dominated and 13 plots where other tree species dominated. In an intra-habitat comparison, we recorded canopy cover and ground vegetation taxonomic richness among 800 sample plots measuring 1 m^2^ in the extended urbanised forest, which featured abundant alien (308 plots) and native trees (492 plots). We observed decreased taxonomic richness among vascular ground plant species by 40% (inter-habitat) and 20% (intra-habitat) in areas dominated by *A. negundo* compared to areas dominated by native tree and shrub species. An abundance of *A*. *negundo* was accompanied by increased canopy cover. We found a negative relationship between canopy cover and the number of understory herbaceous species. Thus, the interception of light and the restriction of its amount for other species is a main factor supporting the negative influence of *A*. *negundo* on native plant communities.

## Introduction

Ash-leaved maple (*Acer negundo* L.) is an invasive tree in the territory of Northern Eurasia that is currently colonising disturbed and semi-natural territories^1–4^. *A*. *negundo* actively restores itself in the urbanised forests of the Middle Urals^5,6^, but the invasion of ash-leaved maple is dangerous for some types of plant communities^7,8^. In communities dominated by *A*. *negundo*, the diversity of native plants decreases^7,9,10^. Invasion of the European *Acer platanoides* L.^11^ and the Far Eastern *Acer ginnala* Maxim produce a similarly negative effect on the diversity of native plant communities in North America^12^.

The ash-leaved maple is not only an alien and invasive species but also a transformer species. Transforming species significantly change the conditions in the invaded ecosystems^13^. The impact of transformer species is realised by influences on the light regime of communities^12,14–16^, nutrient cycles^12,17–21^ and different components of the biota^22–27^. Not all alien species achieve high abundance in invaded communities. Therefore, not all alien species significantly affect local ecosystems. Most of the alien plants are present with a low abundance in limited types, as a rule, of highly anthropogenically transformed habitats and therefore are not considered as transformers or invasive, but as naturalized species. So, when studying the ecological characteristics of alien plants, the features of transformer species arouse more interest versus both native and alien, but not actively spreading, species.

Light is an essential factor determining the productivity^28–30^ and species composition of plant communities^15,31,32^. Competition and specialization in light use in forest communities are especially significant because woody plants exert a substantial environmental influence on all other organisms through shading.

**Figure 1:**
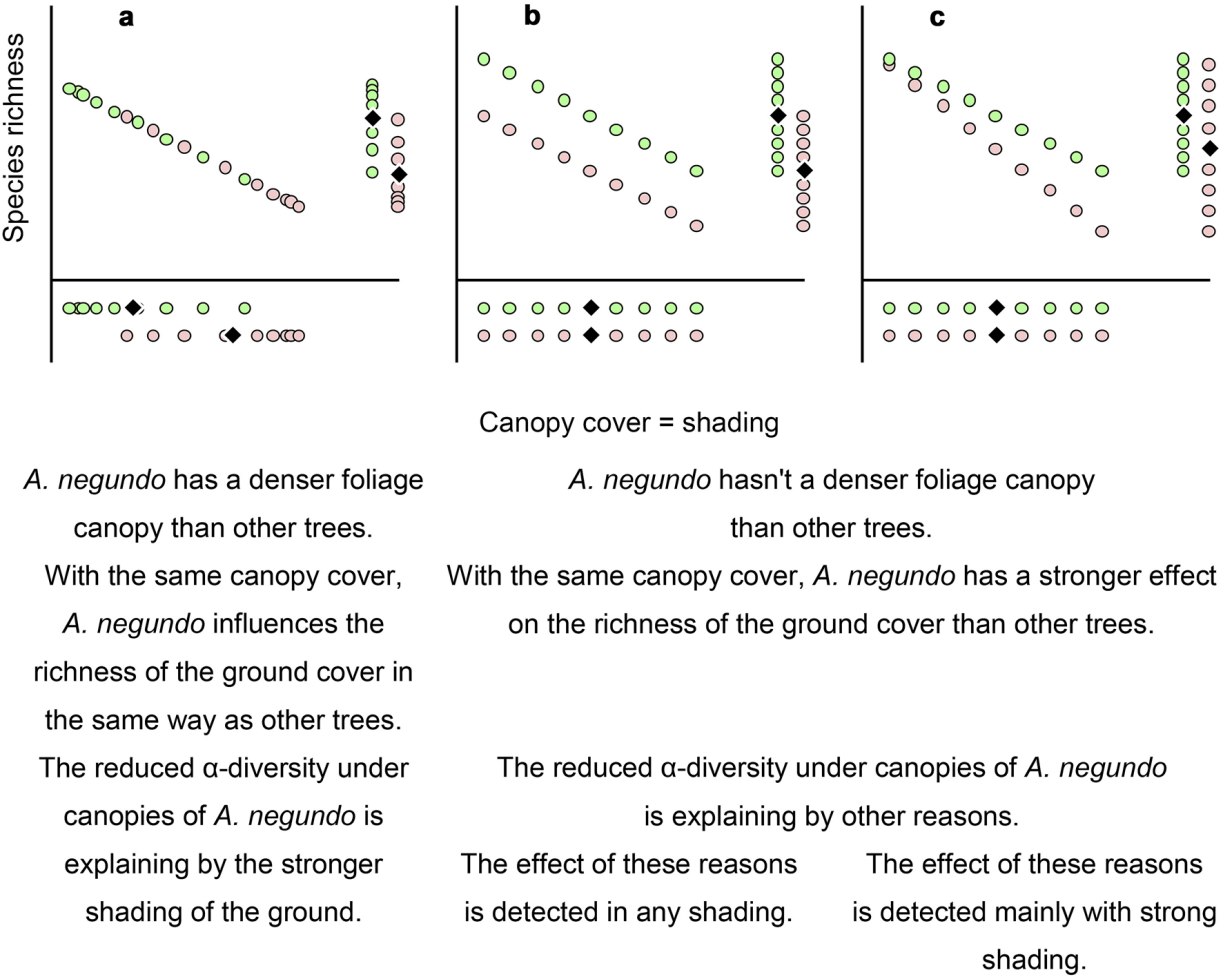
Hypothetical mechanisms of the generation of reduced diversity of the ground cover throughout shading in communities with *Acer negundo* (pink circles) in comparison with other trees (green circles); rhombus—average rates.

Shading is often considered a valid mechanism of the invasive plant influence on native communities^33–35^. This hypothesis seems easy to understand but has no absolute experimental support. Sometimes, higher shading under the invasive plant canopies is not confirmed^36,37^, including under *A. negundo*^8,38^. However, more publications verify that invasive plants form a denser leaf canopy than native ones^33,34,39,40^, including *A. negundo*^40^. So, it can be hypothesized that the ash-leaved maple shades the soil surface more completely than trees of other species (Fig. 1a), implying that the canopy cover in thickets of *A*. *negundo* is denser than in thickets of other trees. However, at the same time, similarly dense canopy cover decreases the richness of ground cover in the same way regardless of the tree species forming the canopy.

In addition to the hypothesis about the strong shading of *A*. *negundo*, it is possible that even with the same canopy cover, the ash-leaved maple has a stronger effect on ground cover than other trees (Fig. 1b,c). This suggestion implies that the reduced richness of the ground cover under *A*. *negundo* is associated not only with shading but also with certain other mechanisms. The reasons for this are potentially very diverse and may include increased litter formation^34^, the different chemical composition of litter^39^ and soils^38^ as well as slower^41^ or accelerated^38^ litter decomposition. Furthermore, direct allelopathic effects of invasive plants on native plants are possible^42–47^, as is the effect of invasive plants on soil organisms^22,23^.

We emphasise the need for further research, including in new geographic regions, to identify the mechanisms of *A*. *negundo* invasion and the impact of this invasion on native plant communities. In this study, we conducted two field investigations to estimate the impacts of ash-leaved maple invasion on native plant communities.

In this study, we focused on testing the hypothesis of whether the associated effects of the canopy cover of *A*. *negundo* can explain its effect on living ground vegetation, namely shrubs and herbs. We hypothesized that the main direction of the relationship between canopy cover and ground cover diversity is negative; that is, with an increase in canopy cover, the number of ground cover species decreases. This paper analyses whether the ability of *A*. *negundo* to create a dense canopy explains its influence on ground cover.

We tested three hypotheses. Firstly, we supposed, that canopy cover in stands dominated by *A. negundo* is higher than canopy cover in stands dominated by other species; Secondly, we surmised that the species richness of ground cover plants is lower in stands dominated by *A. negundo*. Finally, we hypothesized, that species richness of ground cover plants exhibits a negative association with increasing canopy cover that is similar for each invasive A. negundo, alien species, and native species.

In most studies of the ecological features of alien plants, a pairwise comparison of sample plots selected for the presence/absence or dominance/non-dominance of invasive plants located in extended communities and habitats perform^9,10,36,48,49^. However, while using such an experimental design, it is difficult to prove that the established characteristics of the invasive communities are due to the influence of alien plants and not to the original properties of the habitats. To reduce this ambiguity, we used two comparison experimental designs: inter-habitat and intra-habitat comparison.

## Results

### Canopy cover

In both the inter-habitat and intra-habitat comparisons, higher canopy cover was observed for *A*. *negundo* in comparison with other species of woody plants in urban pine forests.

#### Inter-habitat comparison

**Figure 2:**
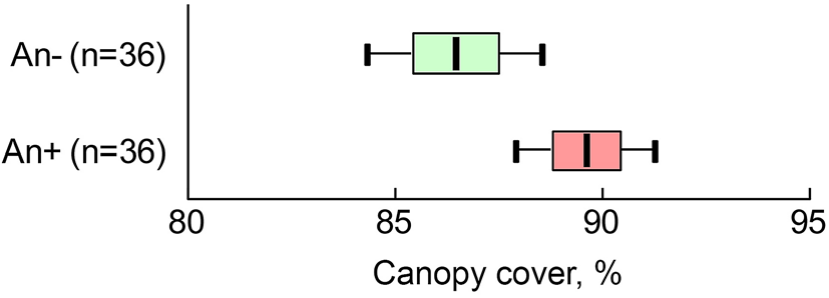
Average rates (± SE, ± 95CI) of canopy cover in communities dominated by *Acer negundo* (An+ ; pink plots) and other tree species (An− ; green plots).

We observed a slightly higher canopy cover in communities dominated by the ash-leaved maple compared to communities with other woody dominants. In ANOVA, with fixed effects "plot type" and "year" and a random effect "site", the differences between plots dominated by invasive *A. negundo* (An +) and dominated by other tree species (An −) in canopy cover were significant (*F*plot type_(1; 54)_ = 11.09; *P* = 0.0016), as well as the effect of the year of observation (*F*year_(2; 54)_ = 3.55; *P* = 0.0356).The interaction of factors with fixed effects was insignificant (*F*plot type × year_(2; 54)_ = 0.33; *P* = 0.7186). The absolute differences in the mean values of canopy cover between the plots were small (Fig. 2): 90 ± 1% in the An+ plots and 86 ± 1% in the An− plots.

#### Intra-habitat comparison

**Figure 3:**
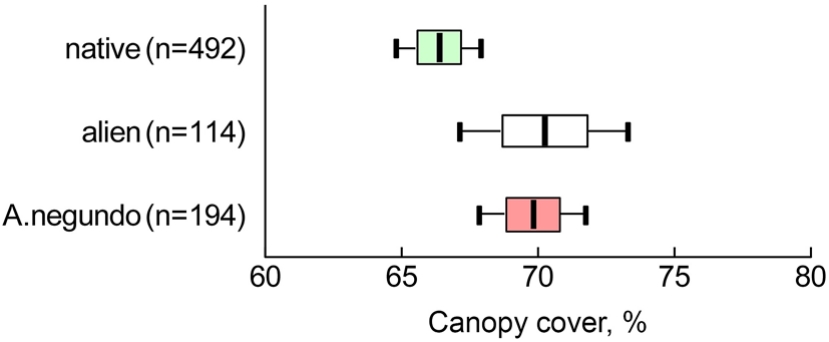
Average rates (± SE, ± 95CI) of canopy cover in areas dominated by native (green plots), alien, excluding *Acer negundo* (white plots) species and individually—*Acer negundo* (pink plots).

Using Moran's I autocorrelation, similar canopy cover values clustered at distances of 10–15 m. Consequently, the heterogeneity of canopy cover distribution covered 2–4 neighboring plots. We found a higher canopy cover in areas dominated by *A*. *negundo* compared to areas with other tree dominants. In the one-way ANOVA, the differences in canopy cover between the plots dominated by native and alien tree species versus *A*. *negundo* were significant (*F*plot type_(2; 797)_ = 4.21; *P* = 0.0151). The absolute differences in the mean values of canopy cover between the options were small (Fig. 3): areas with native dominants showed 66 ± 1%, with *A*. *negundo* 70 ± 1% and with other alien dominants 70 ± 2%. The conclusion about a significant difference in the canopy cover of different tree dominants does not change when regarding the connectivity of points, using ANOVA with mixed effects: with a block size of 5 × 5 m P < 0.0001; with a block size of 10 × 10 m P = 0.0100; with a block size of 20 × 20 m P = 0.0016.

**Figure 4:**
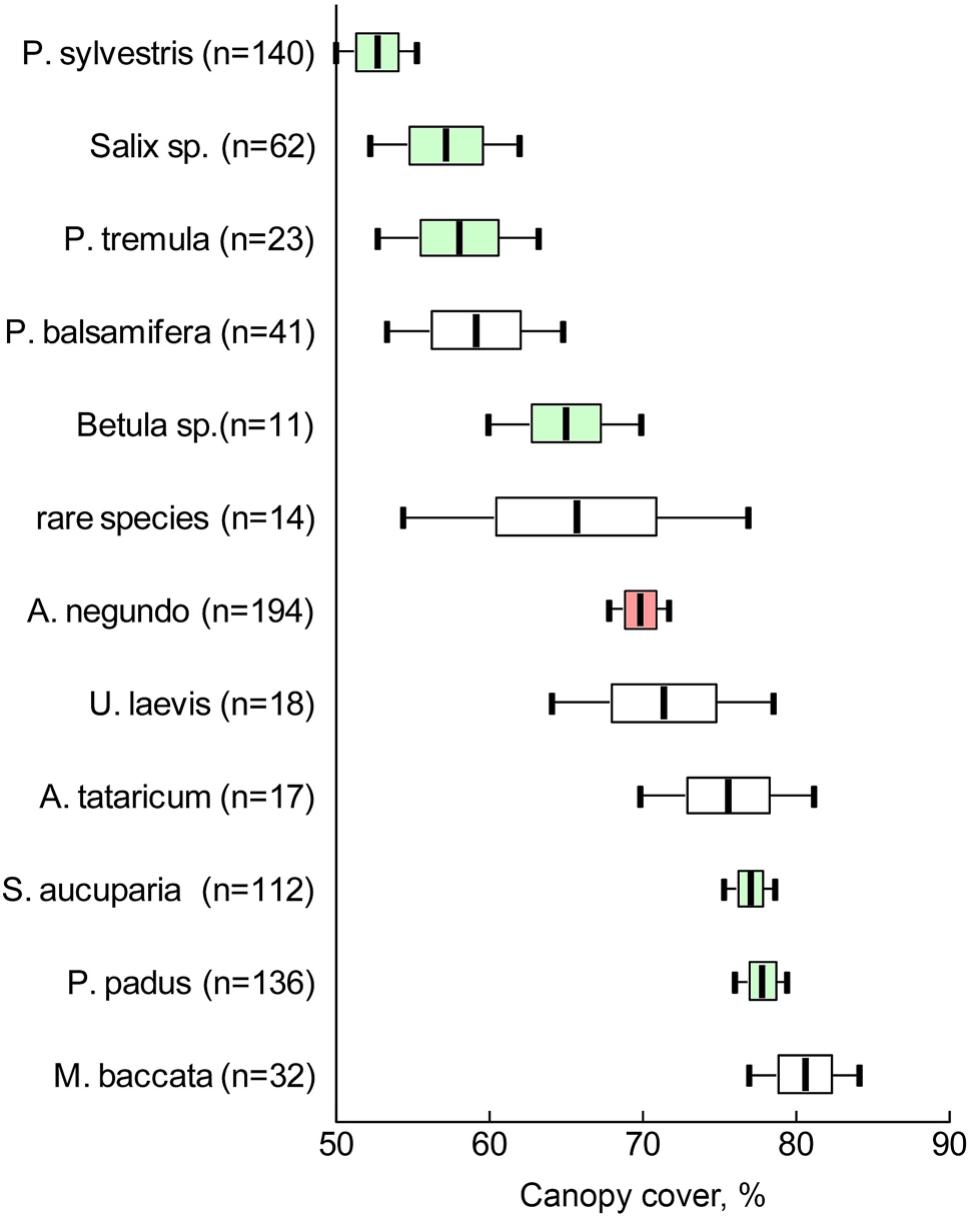
Average rates (± SE, ± 95CI) of canopy cover of different species of native (green plots) and alien (whitr and pink plots) tree species; rare species—the indication for a group of plots dominated by species rarely found in the studied site (*Amelanchier spicata*, *Caragana arborescens*, *Cotoneaster lucidus*, *Crataegus sanguinea*, *Larix sibirica*, *Lonicera xylostella*, *Tilia cordata*).

We found that native and alien woody species resulted in heterogeneous canopy cover (Fig. 4). Some alien species, such as *Populus balsamifera*, had thin canopies, while some native species, such as *Prunus padus* and *Sorbus aucuparia*, had dense canopies. Moreover, among the alien species, the canopies of *A*. *negundo* were not the densest. Some alien woody plants (namely *Ulmus laevis*, *Acer tataricum*, and *Malus baccata*) had higher average canopy cover than *A*. *negundo*.

### Richness of ground cover

In both inter-habitat and intra-habitat comparisons, we observed reduced species richness of the ground cover on plots with a tree layer formed by the ash-leaved maple.

#### Inter-habitat comparison

**Figure 5:**
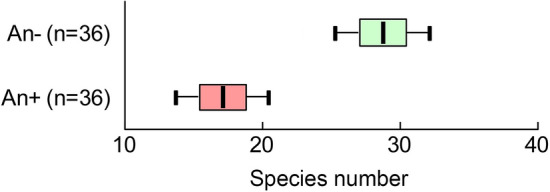
Average rates (± SE, ± 95CI) of species' number per 400 m^2^ in communities dominated by *Acer negundo* (An+ ; pink plots) and other tree species (An− ; green plots).

We observed reduced species richness of the ground cover in communities dominated by *A*. *negundo* compared to communities with other dominant trees. In ANOVA, with fixed effects "plot type" and "year" and a random effect "site", the differences between plots An+ and An− in the number of species per 400 m^2^ were significant (*F*plot type_(1; 54)_ = 63.69; *P* < 0.0001). The observation year (*F*year_(2; 54)_ = 0.30; *P* = 0.7433) and the interaction between the factors of plot type and year of observation were not significant (*F*plot type × year_(2; 54)_ = 1.22; *P* = 0.3031). The absolute differences in average values of species richness of the ground cover between the plot types were significant (Fig. 5): 17.1 ± 1.7 species per 400 m^2^ in the An+ plots and 28.8 ± 1.7 species per 400 m^2^ in the An− plots.

#### Intra-habitat comparison

**Figure 6:**
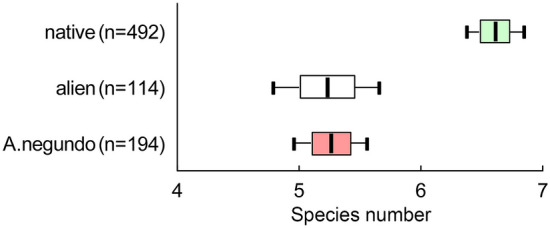
Average number (± SE, ± 95CI) of observed ground cover species per m^2^ on plots dominated by native (green plots), alien, excluding *Acer negundo* (white plots) species, and individually—on plots dominated by *Acer negundo* (pink plots).

Using Moran's I autocorrelation, we found similar values of the ground cover richness clustered at distances of 5–10 m; heterogeneities covered up to 3 neighboring sites. We found that on the plots with alien woody dominants, including *A*. *negundo*, the number of ground cover species was lower in comparison to sites with native dominants (*F*plot type_(2; 797)_ = 28.58; *P* < 0.0001). We observed that absolute differences in average values of species richness between the plots with native woody dominants were 6.6 ± 0.1 species per m^2^; with alien woody dominants, excluding *A*. *negundo*,—5.2 ± 0.2 species per m^2^; and with *A*. *negundo—*5.3 ± 0.2 species per m^2^ (Fig. 6). The conclusion about the difference in the richness of ground cover species under the different tree dominant canopies remains highly significant (P < 0.0001) for any block size (5 × 5, 10 × 10, or 20 × 20 m) in an ANOVA with mixed effects regarding the spatial connectivity of plots.

**Figure 7:**
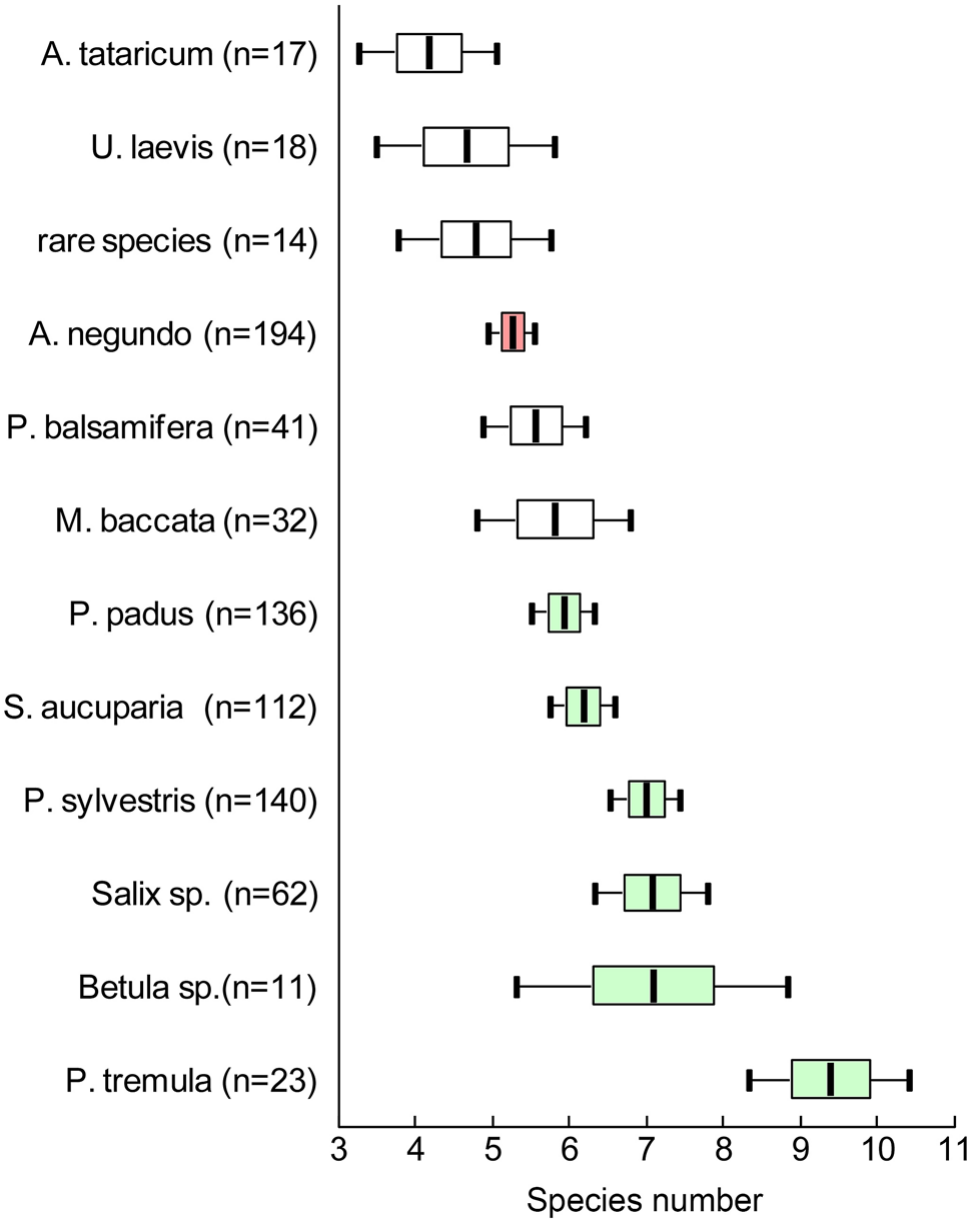
Average number (± SE, ± 95CI) of observed ground cover species per m^2^ on plots under the canopies of different native (green plots) and alien (white and pink plots) tree species; rare species—the indication for a group of plots dominated by species rarely found in the studied site (*Amelanchier spicata*, *Caragana arborescens*, *Cotoneaster lucidus*, *Crataegus sanguinea*, *Larix sibirica*, *Lonicera xylostella*, *Tilia cordata*).

Plots were relatively homogeneous in terms of richness of the ground layer under the canopy regardless of whether the dominant species was native or alien (Fig. 7). Areas dominated by different species of alien plants did not differ in terms of ground richness. Among the native plants, two groups of species were distinguished according to the richness of ground cover communities: 1) underbrush shrubs under which the species richness of ground cover was not the highest—*Prunus padus* and *Sorbus aucuparia*, and 2) trees of the first and second tiers under which was observed the highest species richness of ground cover—*Pinus sylvestris*, *Salix* sp., *Betula* sp., *Populus tremula*.

### Relationship between canopy cover and species richness of ground cover

In both the inter-habitat and intra-habitat comparisons, a negative correlation was found between canopy cover and the number of vascular plant species comprising ground cover.

#### Inter-habitat comparison

**Figure 8:**
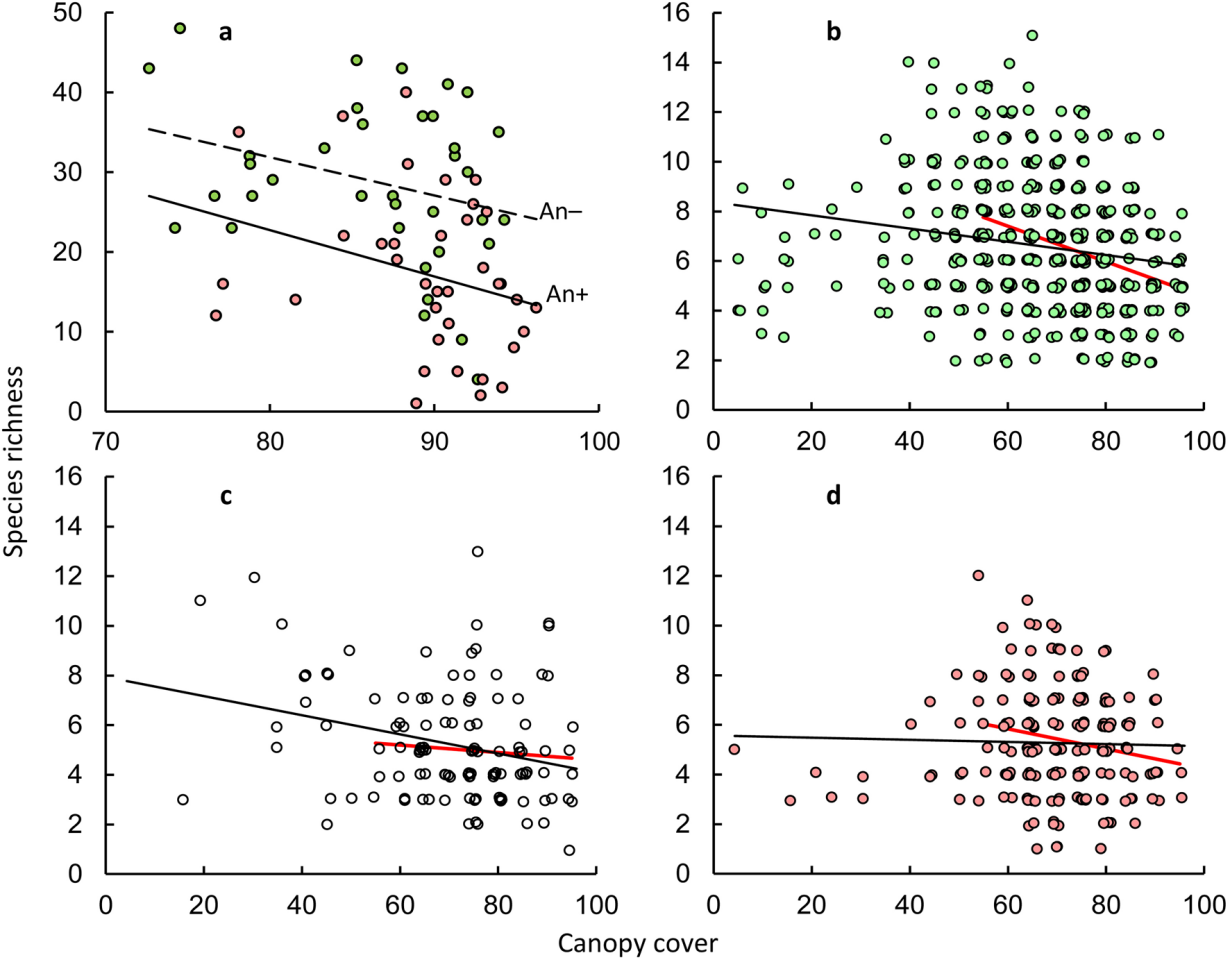
Relationship between the average canopy cover and the number of ground cover species per 400 m^2^ (inter-habitat comparison) (**a**) and per 1 m^2^ (intra-habitat comparison) at plots dominated by native tree species (green circles) (**b**), alien tree species, excluding *Acer negundo* (white circles) (**c**), and individually—*Acer negundo* (pink circles) (**d**). The points are slightly jittered to avoid overlapping on Figures b–d. The approximations in the range of 55–95% canopy cover in Figures b–d are shown with red lines.

In ANCOVA with fixed effects "plot type", "canopy cover" and "year", the species richness of the ground cover significantly depended only on the effects "plot type" (*F*plot type_(1; 60)_ = 13.61; *P* = 0.0005) and "canopy cover" (*F*cover_(1; 60)_ = 6.02; *P* = 0.0170). No other effects, including interaction effects, were significant. This means that the angle of inclination of the lines (coefficients b in the equation y = a + bx) describing the relationship between canopy cover and the number of species of ground cover on plots dominated by the ash-leaved maple and other tree species did not differ (Fig. 8a). An increase of 10% in canopy cover induces a decrease in the number of ground cover species by 5.82 ± 3.30 species per 400 m^2^ (*P* = 0.0866) in the An+ plots and by 4.77 ± 2.66 species per 400 m^2^ (*P* = 0.0820) in the An− plots.

#### Intra-habitat comparison

Despite the spatial autocorrelation of the parameters, the statistical significance of the relationships between canopy cover and the richness of the ground cover differed little in Pearson correlation and spatial correlation. Under native plant canopies (Fig. 8b) *r* =  − 0.18, conventional *P* < 0.0001, Dutilleul corrected *P* = 0.0002. Under alien plant canopies (Fig. 8c) *r* =  − 0.27, conventional *P* = 0.0035, Dutilleul corrected *P* = 0.0128. Under canopies of *A. negundo* (Fig. 8d) *r* =  − 0.0234, conventional *P* = 0.7465, Dutilleul corrected *P* = 0.7471.

In ANCOVA with the factors "plot type" and "canopy cover", the number of ground cover species on the site significantly depended only on the main effects *F*plot type_(2; 794)_ = 25.73 (*P* < 0.0001) and *F*cover_(1; 794)_ = 12.43 (*P* = 0.0004). The "plot type × canopy cover" interaction was not significant: *F*plot type × cover_(2; 794)_ = 1.69; *P* = 0.1855; this indicates that we did not establish a difference in the angle of inclination of the lines describing the relationship between the canopy cover and the number of ground cover species per m^2^ on plots with different woody dominants (Fig. 8a). However, the species richness of the ground cover changed with the growth of the canopy cover depending on the dominant plants. A significant decrease in species richness with an increase of canopy cover was observed in plots with a dominance of both native (with a 10% increase in canopy cover by 0.27 ± 0.07 species per m^2^; *P* < 0.0001) and alien plants (with a 10% increase in canopy cover by 0.38 ± 0.13 species per m^2^; *P* = 0.0035). On plots dominated by *A. negundo* in the whole variety of canopy cover (5–95%), the number of ground cover species did not change significantly with an increase in canopy cover. With an increase in canopy cover of 10%, the number of ground cover species per m^2^ decreased only by 0.04 ± 0.11; *P* = 0.7465.

Using breakpoint linear regression, we found that the shading effect on terrestrial plants is especially appeared with reaching 55–60% values of total canopy cover. For the relationship "canopy cover—species richness", the ordinates of the breakpoints of linear regression on plots dominated by native tree plants were 57 ± 4% of canopy cover. On plots dominated by alien plants ordinates of the breakpoints were 55 ± 15% of canopy cover, on plots dominated by *A. negundo—*55 ± 7%. With this in mind, Fig. 8b–d additionally show lines describing the change in the richness of the ground cover in the range of 55–90% of canopy cover. In this range, a noticeable decrease in species richness on plots dominated by native plants was observed (with an increase of canopy cover by 10%—by 0.72 ± 0.11 species per 1 m^2^;* P* < 0.0001) and plots with *A. negundo* (with an increase of canopy cover by 10%—by 0.40 ± 0.17 species per 1 m^2^;* P* = 0.0190).

## Discussion

Our results show the general consistency of the appearance at the two spatial scales we observed. We found that in the results obtained both for inter-habitat and intra-habitat comparisons, assessments of the light regime transformation were generally similar. So, in both studies, we confirmed that the canopy cover of *A*. *negundo* is higher than the canopy covers of other tree species. Additionally, we observed a decrease in ground layer species richness linked with an increase in canopy cover. In the inter-habitat comparison, this effect was detected both in thickets of ash-leaved maple and in thickets of other tree species. In our intra-habitat comparison, this effect was traced under the canopies of native and alien trees but not under the canopies of *A*. *negundo*.

Our first working hypothesis supposed that *A*. *negundo* can create strong shading. The ash-leaved maple seems to produce a denser canopy than many other woody species. This thesis cannot be strictly proven by the inter-habitat comparison because we purposefully selected plots in pairs of communities An+ and An− with high and equal canopy cover. However, the intra-habitat comparison also clearly shows that the average cover of *A*. *negundo* is greater. In intra-habitat comparison, estimates of canopy cover under different types of trees and shrubs were gathered randomly. However, the ash-leaved maple does not form the highest canopy cover of leaves present in this study. There are several native and alien plants that form denser canopies. Therefore, it is more correct to summarise our results as the following: *A*. *negundo* creates a canopy of leaves which is equally as dense as the canopy of other alien trees but denser than that of native trees.

This fact could have several explanations. For example, the ability to show varying levels of shading is connected with the different heights of the lower canopy edge. According to our estimates, the higher canopies shade the soil surface at the vertical projection point less than lower canopies: trees of the first layer (n = 218) yielded shading of 55 ± 1%; of the second layer (*A*. *negundo* [n = 194] yielded shading of 70 ± 1%; other species (n = 131) yielded shading of 67 ± 1%); and shrubs (n = 257) yielded shading of 77%. Furthermore, we suggest that high canopy cover formed by alien plants may have a different structure for leaf canopy, with different degrees of leaf overlap or of damage to leaves by phytophages^50–52^.

The conclusion that the ash-leaved maple forms a denser canopy of leaves than native trees is consistent with most of the published data about this species^8,38^. This conclusion is relative to some other invasive plants^33,34,39,40^.

Our second working hypothesis supposed that under the canopy of A. negundo, the species richness of the ground cover is lower than under other alien or native tree species canopy. In both inter-habitat and intra-habitat comparisons, we registered a reduced ground cover species richness under canopies of ash-leaved maple. This result confirms the generally accepted idea that alien plants produce a negative impact on the diversity of native communities. Our earlier inter-habitat comparison showed a decrease in alpha diversity influenced by *A. negundo* of about 40%^10^. However, unlike the conclusions reached through inter-habitat comparison^7,9,10,36,48,49^, the 20% decrease in the diversity of ground cover in our intra-habitat comparison can be linked with the traits of the alien plants rather than with the traits of the habitats they invade.

Our third hypothesis supposed that with an increase in shading level, the number of grass species equally decreases both under *A*. *negundo* and under other tree species. In our inter-habitat comparison, an increase in canopy cover was linked with a decrease in plant diversity both under ash-leaved maple shading and that of other trees. However, the intra-habitat comparison illustrated an increase in the canopy cover of *A*. *negundo* that was not accompanied by a decrease in plant diversity.

**Figure 9:**
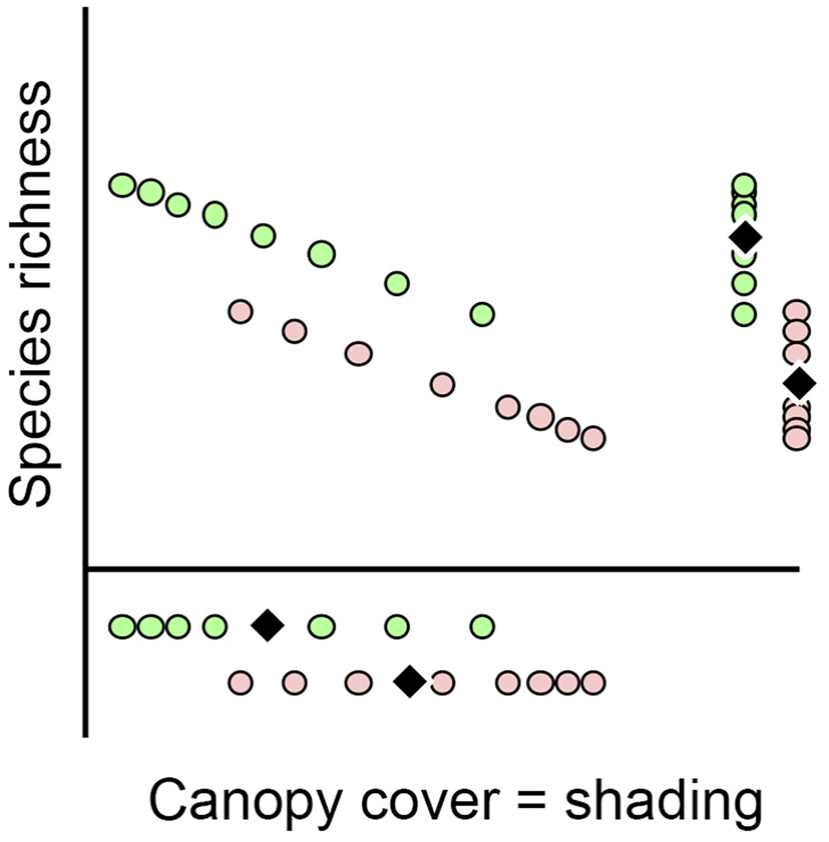
A probable mechanism of the generation of reduced diversity of the ground cover throughout canopy shading in communities dominated by *Acer negundo* (pink circles) in comparison with other trees (green circles); rhombus—average rates.

However, on both spatial scales, the number of grass species under crowns of *A*. *negundo* was less than under crowns of native trees over the whole range of canopy cover. Consequently, the transformation of the light regime of communities does not explain the decrease in plant richness in *A*. *negundo* thickets (Fig. 9). We cannot confirm that our results describe all possible effects of light regime transformation under an ash-leaved maple canopy. Notably, in the Middle Urals, the maximum sun height above the horizon during the summer season is 43–56°. Although the photos shot vertically upward show the canopy cover at the point the camera was located, they do not fully describe the lighting conditions for each plot because they ignore the intensity of side light flux. The difference between the estimates of canopy cover and illumination level may be associated with the preservation of autocorrelation for estimates of the ground cover species richness after considering the effects of canopy cover (see Supplementary file 4). The difference between the estimates of canopy cover and illumination may be especially large for the intra-habitat comparison, but this difference is smaller for the inter-habitat comparison. One more possible consequence of the transformation of light conditions under the canopy of *A*. *negundo* is a specific change in the spectral composition of light.

Along with a decrease in illumination, the mechanisms of the suppression of ground cover under the influence of the ash-leaved maple may be different, depending on spatial canopy structure. For example, the fact that *A*. *negundo* has a dense and low crown may not only decrease illumination in *A*. *negundo* thickets but also blocks the flow of seeds or other plant diaspores. It is known that the diversity and pool of seed banks may decrease in native communities invaded by alien plants^18,53^, including ash-leaved maple^6^.

In addition, the ability of alien species to reduce diversity via allelopathic effects is often discussed^42,47^, and allelopathic activity has been confirmed for the water extracts from *A*. *negundo* leaves^54,55^ and for the soil from its thickets^56^. However, in another experiment, the allelopathic activity of the soil from the thickets of *A*. *negundo* was not confirmed^27^. Accompanying the direct allelopathic effects on plants of substances secreted by *A*. *negundo*, indirect allelopathy is possible and is a probable effect of *A*. *negundo* on native plants through its primary effect on soil microorganisms. In this experiment, though we did not confirm the effect of soil from ash-leaved maple thickets on seed germination, we observed suppression of mycorrhiza in model grasses^27^. Consequently, direct and indirect allelopathy are possible mechanisms for the influence of *A*. *negundo* on native plants, although their ecological impact requires further evaluation.

## Conclusion

We observed decreased ground cover plant species in large (inter-habitat comparison) and small (intra-habitat comparison) vegetation areas dominated by the alien maple *Acer negundo* compared to areas dominated by native tree and shrub species. At the same time, the dominance of the ash-leaved maple was accompanied by a recorded higher canopy cover of a height of 1–1.2 m. Additionally, in both studies, we established a negative correlation between the canopy cover of trees and the number of vascular plant species of ground cover. Thus, the capture of light and restriction of its amount for other species is a central mechanism that causes several of the negative effects of *A*. *negundo* on native communities. Similar to other studies^36^, we found that high canopy cover, which produces high shading, is not the only mechanism by which *A*. *negundo* realises its potential as a transformer species. Most likely, the ash-leaved maple affects native plants and the structure of communities via additional factors. We believe that further studies are needed to understand which of these mechanisms (influence on the seed bank; direct allelopathy; influence on soil microorganisms; transformation of the physicochemical properties of soil) can explain the impact of *Acer negundo* on the diversity and composition of plant communities.

## Methods

### Study area

The data was collected in urbanised habitats in the southern taiga subzone of the boreal zone of the Middle Urals, located in the territory of the Yekaterinburg urban agglomeration. The average annual temperature is + 3 °C, which has shown an upward trend (1 °C increase in the last 25 years). The average annual precipitation is 542 mm. The average height of snow cover in February is 20–25 cm, and the average duration of snow cover is 160–180 days. Yekaterinburg is a large city with a population of 1.5 million people. The surrounding territories are dominated by pine forests of natural origin on sod-podzolic soils and brown soils. The territory of Yekaterinburg is heavily polluted due to a large number of industrial enterprises and the high density of the transport network. The climate is moderately continental with cold winters and warm summers. *A*. *negundo* actively restores itself in the urbanised forests of the Middle Urals^5,57^. To test our working hypotheses, we executed two studies: the first was an inter-habitat comparison, while the second was carried out as an intra-habitat comparison.

### Inter-habitat comparison

#### Sample plots

The inter-habitat comparison was carried out according to a randomized block design with repeated measurements in time 13 sites of intra-urban vegetation located in the city of Yekaterinburg and its suburbs in the Middle Urals, Russia. The area of the site allowed the placement of at least two plots measuring 20 × 20 m. At each site, we selected two plots, the first in the thickets dominated by ash-leaved maple and the second in thickets dominated by other tree species. A tree species was recognized as dominant if its stem number per the sample plot was the largest. Additionally, its stem number was more than 30% of the total stem number per the sample plot. Thus, two plots in one area formed a linked pair of one plot with the studied impact of *A*. *negundo*; (An +) and one without its impact, (An− ; factor ‘plot type’). Paired plots were located as close together as possible and no further than 0.4 km from each other. Moreover, they were similar by a height above sea level and location relative to human housing and infrastructure, and they had close values in terms of canopy cover. The studied communities occupied habitats whose area ranged from 0.08. to 42.26 ha. The distance from the studied communities to the nearest buildings was from 10 to495 m; from automobile roads—from 10 to 280 m. Most of the sites were located on smooth hillsides. Three sites were situated in local depressions with stagnant moisture. The proportion of the ash-leaved maple stems varied from 35 to 100% (average—74%) in An+ plots; from 0 to 27% (average—12%) in An− plots. According to the European nature information system (EUNIS) habitat classification, 10 sites were classified as small green spaces completely or almost surrounded by buildings (X22) or roads (X23), three sites as large parks (X11) and one site as low forest or shrubs in wetlands (F9)^58^. In total, we laid 26 plots. An− plots were dominated by *Ulmus laevis* Huds. (three plots), *Pinus sylvestris* L. (three), *Malus baccata* (L.) Borkh (two), *Prunus padus* Mill. (one), *Salix fragilis* L. (one), *Tilia cordata* Mill. (one), *Sorbus aucuparia* L. (one), *Salix alba* L. (one). Geographical coordinates of sample plots and generalised data of their characteristics are given in the supplementary material (see Supplementary file 1).

#### Descriptions of vegetation

We performed descriptions of vegetation every year from 2017–2019. In 2018, the vegetation on one of the sites was destroyed. We therefore laid an extra couple of plots at the another site in 2018. We performed 72 descriptions covering the period from June to August in 2017, 2018 and 2019 (24 descriptions per year). We recorded species richness and the total cover of the aboveground organs of plants in the herbaceous layer (in %). The species richness was measured as the number of species per 400 m^2^.

#### Estimation of canopy cover

Every year in mid-July, we took 10 colour photos of the canopy at each plot. We took the photos in randomly selected places, pointing the camera straight up to a height of 0.8–1.2 m. We used a Lumix DMC-FP2 digital camera (CCD sensor: 1/2.5″/10.3 million pixels/primary colour filter; photo resolution: 3648 × 2736 pixels). In total, we took 720 photos. To prepare images for analysis, we used Adobe Photoshop 11.0 (Adobe System Inc., 2008). Each photo was converted into binary so that crowns, tree trunks and other obstacles to natural sunlight were rendered as black pixels. The open sky was displayed as white pixels. The analysis of canopy cover was performed in Matlab R2018b (9.5.0.944444, The MathWorks Inc., 2018) using the original code. The code and an example of a pre- and post-processing photo of canopies are published in the supplementary material (see Supplementary file 2).

### Intra-habitat comparison

#### Site

The second study was conducted in June 2018 in the Yugo-Zapadny forest park in the city of Yekaterinburg. We performed it as an analysis of vegetation at 800 points, spatially joined in a regular grid located in one habitat type. According to the EUNIS classification, a forest park is an X11 habitat type (large parks)^58^. We laid the site measuring 795 × 20 m on the 7°-northerly slope from the top of a small ridge to the middle of the slope. The site was generally populated by nettle pine forests with an undergrowth of *Rubus idaeus* L., forb-grass and small-grass. The pine stand was strongly disrupted by selective felling, and derivative communities with *Populus balsamifera* L. and *A*. *negundo* in the tree layer were formed at certain places. On a 795 × 20 m site, we laid five parallel transects 795 m in length on each of which 160 plots were marked at 5 m intervals. As a result, we formed a square grid with a step of 5 m and 800 plots in its nodes. Situation of the study site and generalised data of its characteristics are given in the supplementary material (see Supplementary file 1).

#### Characteristics of plant communities and the estimation of canopy cover

We took two photos with the Lumix DMC-FP2 digital camera at each of the 800 plots. The first photo was taken straight up from a height of 0.8–1.2 m, while the second was taken straight down from a height of 1.5–2 m. At the same time, a 1 × 1 m frame was laid on the ground.

In each canopy photo, the total canopy cover as a percentage of the field of view where 100% considered the fully closed canopy, and 0%—the fully opened sky, was visually estimated in 5% increments. We also identified the species or at minimum the genus of trees and shrubs which fell into the frame of each photo. The proportion of coverage of each taxon was estimated by eye. In each photo of the ground cover, we identified the species, the genus or at minimum the family of low shrubs, herbs and individual trees less than 1.5 m in height. We counted only the number of taxa that were located within a 1 × 1 m frame.

During plant identification, we used a list including 131 species based on 27 preliminary geobotanical descriptions of this section of the forest park^6^. We were able to identify some of the plants in the photos only to the family or genus level. At the same time, plants we were unable to identify to the species level were considered as different conditional species if we could distinguish their morphological features. This made it possible to sufficiently fully calculate the total number of vascular plant species on each plot. We used the term ‘species richness’ when evaluating alpha diversity. The community composition is sometimes analyzed from photos to solve particular problems^59,60^.

At each site, we identified a dominant taxon of woody plants without dividing their life forms (shrubs, undergrowth trees or trees of the second and first layers). The taxon which occupied the largest share of the total canopy cover in the image was considered dominant.

In the array of 800 measurements, the tree dominant species' canopy cover relative to the total canopy cover varied from 30 to 100% (average—69% of the total canopy cover). But on some plots, total canopy cover was low, including the dominant taxon canopy cover. Thus, the dominant taxon total canopy cover was less than 10% only at 16 plots, from 10 to 20%—at 26 plots. The number of plots with a low total canopy cover of the dominant was few (total canopy cover of the dominant taxon was less than 20% only on 4.5% of the plots). Therefore, we decided not to exclude such sites from the analysis to preserve the ability to analyze all 800 measurements.All woody plant species encountered were assigned to one of two groups: native (*Betula* spp.; *Crataegus sanguinea* Pall.; *Larix sibirica* Ledeb.; *Lonicera xylosteum* L.; *Prunus padus* L.; *Pinus sylvestris* L.; *Populus tremula* L.; *Salix* spp.; *Sorbus aucuparia* L.; *Tilia cordata* Mill.; *Viburnum opulus* L.) or alien (*Acer negundo* L.; *Acer tataricum* L.; *Amelanchier spicata* (Lam.) K. Koch; *Caragana arborescens* Lam.; *Cotoneaster lucidus* Schltdl; *Fraxinus pennsylvanica* Marshall; *Malus baccata* (L.) Borkh; *Populus balsamifera* L.; *Ulmus laevis* Huds.).

### Data analysis

During the inter-habitat comparison, we analyzed values of canopy cover and ground cover species richness using a mixed ANOVA model with fixed effects "plot type" and "year of observation" and with an additional random effect "site" to take into account the spatial connectivity of areas An+ and An− . The link of canopy cover and the number of ground cover species in the inter-habitat comparison we assessed using ANCOVA with discrete factors "plot type" and "year" and the continual factor "canopy cover". Additionally, we used the breakpoint regression to determine whether the species richness of the terrestrial layer changes in the same or different way with increasing shading at high and low levels of crown density. The breakpoint regression was made as a combination of two rays with different inclines, connecting each other at a breakpoint. For intra-habitat comparison, we analyzed the canopy cover values using one-way ANOVA with the factor "plot type". The ground cover species number analyzed using one-way ANOVA with the factor "plot type" and ANCOVA with the discrete factor "plot type" and the continual factor "canopy cover".

In our experimental design of intra-habitat comparison, the appearance of effects related to the spatial interconnection of measures was possible. Therefore, we rechecked some of the results obtained by standard ANOVA, ANCOVA correlation analysis methods using spatial statistical methods. We calculated the spatial autocorrelation (Moran's I) of the parameters. To determine whether the spatial connectivity of the points affects the mean trait values (canopy cover and species richness of ground cover), we used a mixed ANOVA model with a fixed effect "plot type" and with an additional random effect "block". We defined the block as a group of nearby points placed in 5 × 5, 10 × 10, or 20 × 20 m squares. Additionally, we used Dutilleul correction when calculating the correlation between canopy cover and the ground cover species richness.

For the percentage of canopy cover, we subjected the values to arcsine transformation. The values of the quantities presented in the text are the mean values of the features ± standard errors. In all cases, the text indicates the results of *F*-tests for fixed effects obtained in full ANOVA / ANCOVA models without excluding individual factors or their interactions. In some cases, when the main factor effects or the interaction of factors were not significant, the results of the *F*-tests are not shown in the manuscript. The full description of ANOVA / ANCOVA models are presented in the Supplementary material (see Supplementary file 3). The calculations performed in the JMP 10.0.0 (SAS Institute Inc., USA, 2012), STATISTICA 8.0 (StatSoft Inc., USA, 1984–2007), and PASSAGE 2^61^.

## Supplementary Information

Supplementary Information 1.

Supplementary Information 2.

Supplementary Information 3.

Supplementary Information 4.

## Data Availability

The datasets generated and/or analysed during the current study are available from the corresponding author on reasonable request.
