# Supplementary material for: High canopy cover of invasive Acer negundo L. affects ground vegetation taxonomic richness
Source: Sci Rep. 2021 Oct 21;11:20758. doi: 10.1038/s41598-021-00258-x (PMC8531358; doi:10.1038/s41598-021-00258-x)
Supplement: Supplementary file 1 — Supplementary Information 1. [file 41598_2021_258_MOESM1_ESM.pdf]

Supplementary materials for the paper "High canopy cover of invasive *Acer negundo* L. affects ground vegetation taxonomic richness"

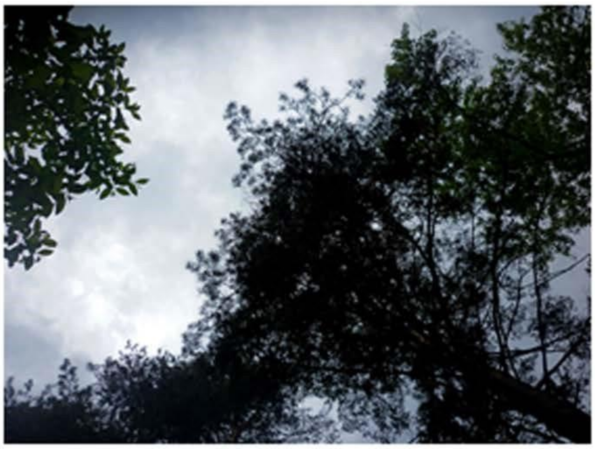

a

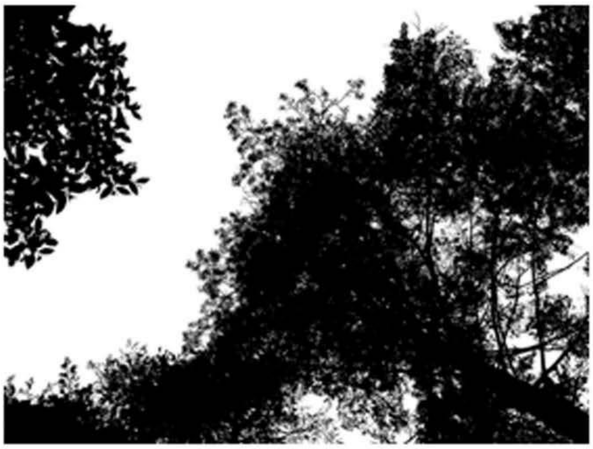

b

Figure 1. Sample of the photo before processing in Adobe Photoshop (a) and processed binary image (b).

Below is the script for canopy cover (percentage of black pixels on the binary photo of canopies) estimating for software package MATLAB. Estimation performed using MATLAB R2018b (9.5.0.944444, The MathWorks Inc., 2018).

```
dirlist = dir('Folder\*.jpg');
for k = 1:length(dirlist);
fname = dirlist(k).name;
[path,name,ext] = fileparts(fname);
a = imread(fullfile('Folder\', fname));
w = 255;
b = 0;
Somk = length(find(a<=b))/(length(find(a>=w))+length(find(a<=b)));
table(k,2) = c_cover
alternative{1,1} = 'c_cover'
alternative{2,1} = 'fname'
alternative{1,k+1} = c_cover
alternative{2,k+1} = fname
end
```
