# Supplementary material for: High canopy cover of invasive Acer negundo L. affects ground vegetation taxonomic richness
Source: Sci Rep. 2021 Oct 21;11:20758. doi: 10.1038/s41598-021-00258-x (PMC8531358; doi:10.1038/s41598-021-00258-x)
Supplement: Supplementary file 2 — Supplementary Information 2. [file 41598_2021_258_MOESM2_ESM.docx]

Sample plot An+_1 was classified by EUNIS Habitat classification as X22. Coordinates - N 56 ̊ 41'18,03'' E 60 ̊ 50'11,69''. Observed tree species: *Acer negundo* L., *Padus avium* Mill. Tree dominant - *Acer negundo* L. Observed number of herbal species in 2017 is 40, in 2018 - 35, in 2019 - 37. Average canopy cover recorded on a height 1-1,2 m is 83,62 %.

Sample plot An+_2 was classified by EUNIS Habitat classification as X23. Coordinates - N 56 ̊ 49'6,59'' E 60 ̊ 35'47,64''. Observed tree species: *Acer negundo* L., *Fraxinus pennsylvanica* Marsh., *Malus baccata* (L.) Borkh., *Padus avium* Mill., *Populus balsamifera* L. Tree dominant - *Acer negundo* L. Observed number of herbal species in 2017 is 5, in 2018 - 2, in 2019 - 1. Average canopy cover recorded on a height 1-1,2 m is 91,06 %.

Sample plot An+_3 was classified by EUNIS Habitat classification as X11. Coordinates - N 56 ̊ 45'15,3'' E 60 ̊ 49'4,48''. Observed tree species: *Acer negundo* L., *Betula pendula* Roth, *Malus baccata* (L.) Borkh., *Padus avium* Mill. Tree dominant - *Acer negundo* L. Observed number of herbal species in 2017 is 10, in 2018 - 5, in 2019 - 3. Average canopy cover recorded on a height 1-1,2 m is 93,00 %.

Sample plot An+_4 was classified by EUNIS Habitat classification as X22. Coordinates - N 56 ̊ 41'48,08'' E 60 ̊ 48'5,95''. Observed tree species: *Acer negundo* L., *Malus baccata* (L.) Borkh., *Padus avium* Mill. Tree dominant - *Acer negundo* L. Observed number of herbal species in 2017 is 4, in 2018 sample plot was destroyed and we replace it to An+_13 sample plot. Average canopy cover recorded on a height 1-1,2 m is 92,96 %.

Sample plot An+_5 was classified by EUNIS Habitat classification as F9.2. Coordinates - N 56 ̊ 41'30,63'' E 60 ̊ 48'23,34''. Observed tree species: *Acer negundo* L., *Padus avium* Mill., *Sorbus aucuparia* L. Tree dominant - *Acer negundo* L. Observed number of herbal species in 2017 is 16, in 2018 - 13, in 2019 - 13. Average canopy cover recorded on a height 1-1,2 m is 93,45 %.

Sample plot An+_6 was classified by EUNIS Habitat classification as X23. Coordinates - N 56 ̊ 49'6,53'' E 60 ̊ 35'43,08''. Observed tree species: *Acer negundo* L., *Malus baccata* (L.) Borkh., *Padus avium* Mill. Tree dominant - *Acer negundo* L. Observed number of herbal species in 2017 is 22, in 2018 - 18, in 2019 - 25. Average canopy cover recorded on a height 1-1,2 m is 92,21 %.

Sample plot An+_7 was classified by EUNIS Habitat classification as X22. Coordinates - N 56 ̊ 47'53,72'' E 60 ̊ 35'43,08''. Observed tree species: *Acer negundo* L., *Malus baccata* (L.) Borkh., *Padus avium* Mill., *Ulmus laevis* Pall. Tree dominant - *Acer negundo* L. Observed number of herbal species in 2017 is 26, in 2018 - 14, in 2019 - 15. Average canopy cover recorded on a height 1-1,2 m is 92,54 %.

Sample plot An+_8 was classified by EUNIS Habitat classification as X22. Coordinates - N 56 ̊ 53'35,84'' E 60 ̊ 36'6,44''. Observed tree species: *Acer negundo* L., *Betula pendula* Roth, *Malus baccata* (L.) Borkh., *Querqus robur* L. Tree dominant - *Acer negundo* L. Observed number of herbal species in 2017 is 11, in 2018 - 24, in 2019 - 31. Average canopy cover recorded on a height 1-1,2 m is 90,43 %.

Sample plot An+_9 was classified by EUNIS Habitat classification as X11. Coordinates - N 56 ̊ 47'0,45'' E 60 ̊ 34'8,65''. Observed tree species: *Acer negundo* L., *Padus avium* Mill., *Pinus sylvestris* L., *Tilia cordata* Mill., *Sorbus aucuparia* L. Tree dominant - *Acer negundo* L. Observed number of herbal species in 2017 is 21, in 2018 - 21, in 2019 - 22. Average canopy cover recorded on a height 1-1,2 m is 86,30 %.

Sample plot An+_10 was classified by EUNIS Habitat classification as X23. Coordinates - N 56 ̊ 47'59,76'' E 60 ̊ 36'15,16''. Observed tree species: *Acer negundo* L., *Crataegus sanguinea* Pall., *Fraxinus pennsylvanica* Marsh., *Malus baccata* (L.) Borkh., *Padus avium* Mill., *Querqus robur* L. Tree dominant - *Acer negundo* L. Observed number of herbal species in 2017 is 16, in 2018 - 16, in 2019 - 15. Average canopy cover recorded on a height 1-1,2 m is 91,42 %.

Sample plot An+_11 was classified by EUNIS Habitat classification as X11. Coordinates - N 56 ̊ 47'29,59'' E 60 ̊ 34'53,82''. Observed tree species: *Acer negundo* L., *Malus baccata* (L.) Borkh., *Padus avium* Mill., *Sorbus aucuparia* L., *Ulmus laevis* Pall. Tree dominant - *Acer negundo* L. Observed number of herbal species in 2017 is 16, in 2018 - 14, in 2019 - 12. Average canopy cover recorded on a height 1-1,2 m is 78,49 %.

Sample plot An+_12 was classified by EUNIS Habitat classification as X22. Coordinates - N 56 ̊ 53'23,29'' E 60 ̊ 36'31,46''. Observed tree species: *Acer negundo* L., *Crataegus sanguinea* Pall., *Salix alba* L. Tree dominant - *Acer negundo* L. Observed number of herbal species in 2017 is 19, in 2018 - 29, in 2019 - 29. Average canopy cover recorded on a height 1-1,2 m is 90,32 %.

Sample plot An+_13 was classified by EUNIS Habitat classification as X22. Coordinates - N 56 ̊ 50'46,42'' E 60 ̊ 38'49,25''. One tree spacies was observed - *Acer negundo* L. Observed number of herbal species in 2018 is 8, in 2019 - 9. Average canopy cover recorded on a height 1-1,2 m is 92,56 %.

Sample plot An-_1 was classified by EUNIS Habitat classification as X22. Coordinates - N 56 ̊
41'18,93'' E 60 ̊ 50'8,29''. Observed tree species: *Acer negundo* L., *Crataegus sanguinea* Pall., *Malus baccata* (L.) Borkh., *Populus balsamifera* L., *Querqus robur* L., *Sorbus aucuparia* L. Tree dominant - *Populus balsamifera* L. Observed number of herbal species in 2017 is 44, in 2018 - 43, in 2019 - 48. Average canopy cover recorded on a height 1-1,2 m is 77,50 %.

Sample plot An-_2 was classified by EUNIS Habitat classification as X23. Coordinates - N 56 ̊ 49'06,71'' E 60 ̊35'47,95''. Observed tree species: *Acer negundo* L., *Acer tataricum* L., *Betula pendula* Roth, *Larix sibirica* auct., non Ledeb., *Padus avium* Mill., *Tilia cordata* Mill., *Ulmus laevis* Pall. Tree dominant - *Ulmus laevis* Pall. Observed number of herbal species in 2017 is 27, in 2018 - 24, in 2019 - 33. Average canopy cover recorded on a height 1-1,2 m is 90,56 %.

Sample plot An-_3 was classified by EUNIS Habitat classification as X11. Coordinates - N 56 ̊ 45'10,61'' E 60 ̊ 48'56,23''. Observed tree species: *Acer negundo* L., *Betula pendula* Roth, *Malus baccata* (L.) Borkh., *Padus avium* Mill., *Salix caprea* L., *Sorbus aucuparia* L. Tree dominant - *Padus avium* Mill. Observed number of herbal species in 2017 is 12, in 2018 - 14, in 2019 - 9. Average canopy cover recorded on a height 1-1,2 m is 90,24 %.

Sample plot An-_4 was classified by EUNIS Habitat classification as X22. Coordinates - N 56 ̊ 41'50,56'' E 60 ̊ 48'24,01''. Observed tree species: *Acer negundo* L., *Malus baccata* (L.) Borkh., *Padus avium* Mill., *Salix caprea* L. Tree dominant - *Padus avium* Mill. Observed number of herbal species in 2017 is 4. In 2018 sample plot was destroyed and we replace it to An- _13 sample plot. Average canopy cover recorded on a height 1-1,2 m is 92,66 %.

Sample plot An-_5 was classified by EUNIS Habitat classification as F9.2. Coordinates - N 56 ̊ 41'13,36'' E 60 ̊ 48'2,83''. Observed tree species: *Acer negundo* L., *Malus baccata* (L.) Borkh., *Padus avium* Mill., *Salix fragilis* L., *Sorbus aucuparia* L., Tree dominant - *Salix fragilis* L. Observed number of herbal species in 2017 is 36, in 2018 - 40, in 2019 - 43. Average canopy cover recorded on a height 1-1,2 m is 88,58 %.

Sample plot An-_6 was classified by EUNIS Habitat classification as X23. Coordinates - N 56 ̊ 49'5,33'' E 60 ̊ 35'59,22''. Observed tree species: *Acer negundo* L., *Crataegus sanguinea* Pall., *Pinus sylvestris* L., *Sorbus aucuparia* L. Tree dominant - *Pinus sylvestris* L. Observed number of herbal species in 2017 is 23, in 2018 - 25, in 2019 - 27. Average canopy cover recorded on a height 1-1,2 m is 81,04 %.

Sample plot An-_7 was classified by EUNIS Habitat classification as X22. Coordinates - N 56 ̊
48'1,71'' E 60 ̊ 36'59,07''. Observed tree species: *Acer negundo* L., *Fraxinus pennsylvanica* Marsh., *Populus balsamifera* L., *Salix caprea* L., *Sorbus aucuparia* L., *Ulmus laevis* Pall. Tree dominant - *Ulmus laevis* Pall. Observed number of herbal species in 2017 is 20, in 2018 - 24, in 2019 - 26. Average canopy cover recorded on a height 1-1,2 m is 90,74 %.

Sample plot An-_8 was classified by EUNIS Habitat classification as X22. Coordinates - N 56 ̊ 53'37,28'' E 60 ̊36'8,13''. Observed tree species: *Acer negundo* L., *Betula pendula* Roth, *Malus baccata* (L.) Borkh., *Padus avium* Mill., *Populus balsamifera* L., *Querqus robur* L., *Sorbus aucuparia* L., *Tilia cordata* Mill. *Ulmus laevis* Pall. Tree dominant - *Tilia cordata* Mill. Observed number of herbal species in 2017 is 18, in 2018 - 27, in 2019 - 31. Average canopy cover recorded on a height 1-1,2 m is 81,65 %.

Sample plot An-_9 was classified by EUNIS Habitat classification as X11. Coordinates - N 56 ̊ 47'4,98'' E 60 ̊ 34'5,22''. Observed tree species: *Acer negundo* L., *Betula pendula* Roth, *Malus baccata* (L.) Borkh., *Padus avium* Mill., *Pinus sylvestris* L., *Populus tremula* L., *Sorbus aucuparia* L., *Tilia cordata* Mill. Tree dominant - *Pinus sylvestris* L. Observed number of herbal species in 2017 is 30, in 2018 - 35, in 2019 - 37. Average canopy cover recorded on a height 1-1,2 m is 91,97 %.

Sample plot An-_10 was classified by EUNIS Habitat classification as X23. Coordinates - N 56 ̊ 47'59,04'' E 60 ̊ 36'20,74''. Observed tree species: *Acer negundo* L., *Betula pendula* Roth, *Padus avium* Mill., Pinus sibirica Du Tour, *Querqus robur* L., *Sorbus aucuparia* L., *Tilia cordata* Mill., *Ulmus laevis* Pall. Tree dominant - *Ulmus laevis* Pall. Observed number of herbal species in 2017 is 32, in 2018 - 21, in 2019 - 23. Average canopy cover recorded on a height 1-1,2 m is 90,82 %.

Sample plot An-_11 was classified by EUNIS Habitat classification as X11. Coordinates - N 56 ̊ 47'24,51'' E 60 ̊ 34'58,75''. Observed tree species: *Acer negundo* L., *Betula pendula* Roth, *Malus baccata* (L.) Borkh., *Padus avium* Mill., *Pinus sylvestris* L., *Populus tremula* L., *Sorbus aucuparia* L. Tree dominant - *Pinus sylvestris* L. Observed number of herbal species in 2017 is 29, in 2018 - 27, in 2019 - 23. Average canopy cover recorded on a height 1-1,2 m is 81,16 %.

Sample plot An-_12 was classified by EUNIS Habitat classification as X22. Coordinates - N 56 ̊ 53'25,41'' E 60 ̊ 36'40,47''. Observed tree species: *Acer negundo* L., *Salix alba* L., *Sorbus aucuparia* L., *Ulmus laevis* Pall. Tree dominant - *Salix alba* L. Observed number of herbal species in 2017 is 32, in 2018 - 37, in 2019 - 33. Average canopy cover recorded on a height 1-1,2 m is 83,81 %.

Sample plot An-_13 was classified by EUNIS Habitat classification as X23. Coordinates - N 56 ̊ 48'27,91'' E 60 ̊ 38'49''. Observed tree species: *Betula pendula* Roth, *Larix sibirica* auct., non Ledeb., *Padus maackii* (Rupr.) Kom., *Padus virginiana* (L.) Mill., *Pinus sylvestris* L., *Populus deltoides* Marsh.,*Ulmus laevis* Pall. Tree dominant - *Pinus sylvestris* L. Observed number of herbal species in 2018 is 41, in 2019 - 38. Average canopy cover recorded on a height 1-1,2 m is 88,08 %.

You can also use the *kml file for reviewing this information, which is compatible with Google Earth and SAS Planet programs by following this link: <https://doi.org/10.5281/zenodo.4307740>.
