# Supplementary material for: High canopy cover of invasive Acer negundo L. affects ground vegetation taxonomic richness
Source: Sci Rep. 2021 Oct 21;11:20758. doi: 10.1038/s41598-021-00258-x (PMC8531358; doi:10.1038/s41598-021-00258-x)
Supplement: Supplementary file 3 — Supplementary Information 3. [file 41598_2021_258_MOESM3_ESM.docx]

"High canopy cover of invasive *Acer negundo* L. affects ground vegetation taxonomic richness"

ANOVA and ANCOVA results description, discussed in the manuscript

**Canopy cover**

**Inter-habitat comparison**

| Canopy cover; inter-habitat comparison; ANOVA with fixed and random effects; *dF*_error_ = 54 | | | | |
| --- | --- | --- | --- | --- |
| **Dependent variable** | **Predictors (factors)** | ***dF*** | ***F*** | ***P*** |
| Canopy cover | Plot type (fixed effects) | 1 | 11.09 | 0.0016 |
|  | Year (fixed effects) | 2 | 3.55 | 0.0356 |
|  | Plot type × year (fixed effects) | 2 | 0.33 | 0.7186 |
|  | Site (random effect) | 12 | 5.57 | <0.0001 |

**Intra-habitat comparison**

| Canopy cover; intra-habitat comparison; one-way ANOVA; *dF*_error_ = 797 | | | | |
| --- | --- | --- | --- | --- |
| **Dependent variable** | **Predictors (factors)** | ***dF*** | ***F*** | ***P*** |
| Canopy cover | Plot type (fixed effects) | 2 | 4.21 | 0.0151 |

| Canopy cover; intra-habitat comparison; ANOVA with fixed (plot type) and random effect (block 5×5); *dF*_error_ = 558 | | | | |
| --- | --- | --- | --- | --- |
| **Dependent variable** | **Predictors (factors)** | ***dF*** | ***F*** | ***P*** |
| Canopy cover | Plot type (fixed effects) | 2 | 9.98 | <0.0001 |
|  | Block 5×5 (random effect) | 239 | 1.19 | 0.0516 |

| Canopy cover; intra-habitat comparison; ANOVA with fixed (plot type) and random effect (block 10×10); *dF*_error_ = 692 | | | | |
| --- | --- | --- | --- | --- |
| **Dependent variable** | **Predictors (factors)** | ***dF*** | ***F*** | ***P*** |
| Canopy cover | Plot type (fixed effects) | 2 | 4.63 | 0.0100 |
|  | Block 10×10 (random effect) | 105 | 1.66 | 0.0001 |

| Canopy cover; intra-habitat comparison; ANOVA with fixed (plot type) and random effect (block 20×20); *dF*_error_ = 766 | | | | |
| --- | --- | --- | --- | --- |
| **Dependent variable** | **Predictors (factors)** | ***dF*** | ***F*** | ***P*** |
| Canopy cover | Plot type (fixed effects) | 2 | 6.49 | 0.0016 |
|  | Block 20×20 (random effect) | 31 | 1.41 | 0.0697 |

**Richness of ground cover**

**Inter-habitat comparison**

| Richness of ground cover; inter-habitat comparison; ANOVA with fixed and random effects; *dF*_error_ = 54 | | | | |
| --- | --- | --- | --- | --- |
| **Dependent variable** | **Predictors (factors)** | ***dF*** | ***F*** | ***P*** |
| Richness of ground cover | Plot type (fixed effects) | 1 | 63.69 | <0.0001 |
|  | Year (fixed effects) | 2 | 0.30 | 0.7433 |
|  | Plot type × year (fixed effects) | 2 | 1.22 | 0.3031 |
|  | Site (random effect) | 12 | 10.44 | <0.0001 |

**Intra-habitat comparison**

| Richness of ground cover; intra-habitat comparison; one-way ANOVA; *dF*_error_ = 797 | | | | |
| --- | --- | --- | --- | --- |
| **Dependent variable** | **Predictors (factors)** | ***dF*** | ***F*** | ***P*** |
| Richness of ground cover | Plot type (fixed effects) | 2 | 28.58 | <0.0001 |

| Richness of ground cover; intra-habitat comparison; ANOVA with fixed (plot type) and random effect (block 5×5); *dF*_error_ = 558 | | | | |
| --- | --- | --- | --- | --- |
| **Dependent variable** | **Predictors (factors)** | ***dF*** | ***F*** | ***P*** |
| Richness of ground cover | Plot type (fixed effects) | 2 | 10.87 | <0.0001 |
|  | Block 5×5 (random effect) | 239 | 1.30 | 0.0065 |

| Richness of ground cover; intra-habitat comparison; ANOVA with fixed (plot type) and random effect (block 10×10); *dF*_error_ = 692 | | | | |
| --- | --- | --- | --- | --- |
| **Dependent variable** | **Predictors (factors)** | ***dF*** | ***F*** | ***P*** |
| Richness of ground cover | Plot type (fixed effects) | 2 | 14.55 | <0.0001 |
|  | Block 10×10 (random effect) | 105 | 1.69 | <0.0001 |

| Richness of ground cover; intra-habitat comparison; ANOVA with fixed (plot type) and random effect (block 20×20); *dF*_error_ = 766 | | | | |
| --- | --- | --- | --- | --- |
| **Dependent variable** | **Predictors (factors)** | ***dF*** | ***F*** | ***P*** |
| Richness of ground cover | Plot type (fixed effects) | 2 | 25.75 | <0.0001 |
|  | Block 10×10 (random effect) | 31 | 1.93 | 0.0020 |

**Relationship between canopy cover and species richness of ground cover**

**Inter-habitat comparison**

Richness of ground cover; inter-habitat comparison; ANCOVA with fixed effects; *dF*_error_ = 60

| **Dependent variable** | **Predictors (factors)** | **dF** | **F** | **P** |
| --- | --- | --- | --- | --- |
| Richness of ground cover | Plot type | 1 | 13.61 | 0.0005 |
|  | Year | 2 | 0.54 | 0.5866 |
|  | Canopy cover | 1 | 6.02 | 0.0170 |
|  | Plot type × year | 2 | 0.54 | 0.5866 |
|  | Plot type × canopy cover | 1 | <0.01 | 0.9949 |
|  | Year ×canopy cover | 2 | 0.04 | 0.9574 |
|  | Plot type × year × canopy cover | 2 | 0.24 | 0.7898 |

**Intra-habitat comparison**

Richness of ground cover; intra-habitat comparison; ANCOVA with fixed effects; *dF*_error_ = 794

| **Dependent variable** | **Predictors (factors)** | ***dF*** | ***F*** | ***P*** |
| --- | --- | --- | --- | --- |
| Richness of ground cover | Plot type | 2 | 25.73 | <0.0001 |
|  | Canopy cover | 1 | 12.43 | 0.0004 |
|  | Plot type × canopy cover | 1 | 1.69 | 0.1855 |
