# Supplementary material for: High canopy cover of invasive Acer negundo L. affects ground vegetation taxonomic richness
Source: Sci Rep. 2021 Oct 21;11:20758. doi: 10.1038/s41598-021-00258-x (PMC8531358; doi:10.1038/s41598-021-00258-x)
Supplement: Supplementary file 4 — Supplementary Information 4. [file 41598_2021_258_MOESM4_ESM.pdf]

"High canopy cover of invasive *Acer negundo* L. affects ground vegetation taxonomic richness"

Testing the hypothesis about the absence / preservation of autocorrelation of residuals

We investigated a functional relationship between the canopy cover and the number of ground cover species. At the same time, our estimates of crown density and soil cover richness on the intra-habitat comparison scale are not spatially independent. It allowed us to make the following hypothesis. The positive autocorrelation of estimates of ground cover species richness at distances of 5–10 m may be partially explained by a similar positive autocorrelation of canopy cover at distances of 10–15 m. If this applies, the influence of the "canopy cover" factor, the autocorrelation of the ground cover species richness residuals may disappear after excluding. It would mean that the heterogeneity of the distribution of the ground cover species richness is explained well by the heterogeneity of the distribution of the canopy cover.

We have verified this hypothesis.

We used the model residuals after ANCOVA with the factors "plot type" and "canopy cover" described in the section "Relationship between canopy cover and species richness of ground cover", in the subsection "Intra-habitat comparison". We found that the autocorrelation of the residuals estimates of the ground cover species richness was preserved. Autocorrelation was identified in the distance interval of 5–10 m (see Fig. 1). Moran's autocorrelation coefficient (I) at 99 permutations was I = 0.09664 (P = 0.01000). It means that in addition to crown density, there are some other factors that we did not consider and which determine the aggregation of estimates of the ground cover species richness.

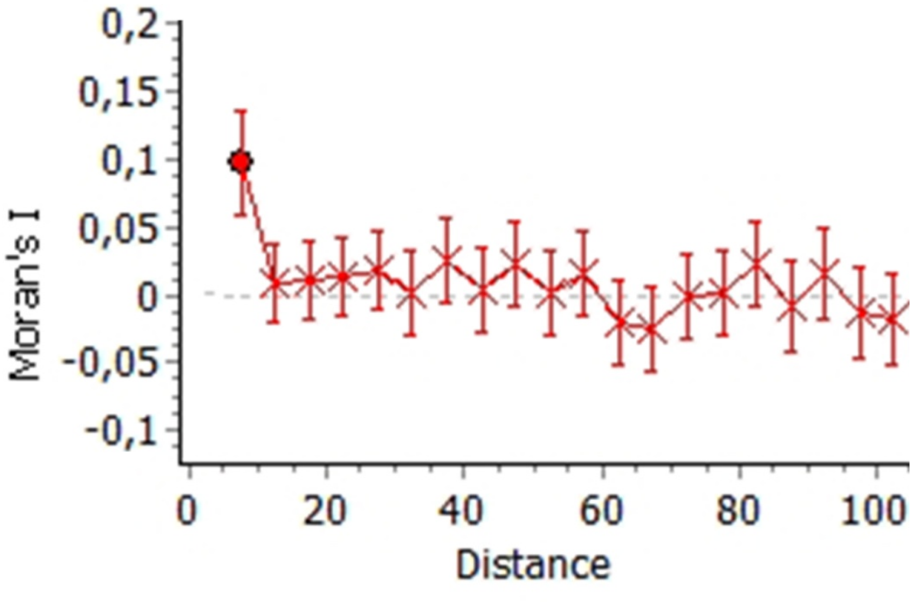

Fig. 1. Moran's (I) values for the residuals of soil cover richness estimates after ANCOVA with the factors "plot type" and "canopy cover"

One of these probable factors may be the disparity in the space between the spots of the distribution of canopy cover (assessed by photos) and spots of shadows falling on the surface, already noted in the text. This disparity can be explained by the low sun angles in our study area,

especially in the spring and autumn seasons, as well as in the morning and evening. Such a disparity of 5-10 m, which is not considered in linear models, such as ANCOVA, seems quite possible.

We note that a specific analysis of the regularities of the spatial distribution parameters of canopies and ground cover was not our main task. However, we believe we can be confident that the spatial connectivity of the observations did not lead to artifacts in our conclusions.
